# Adaptive Cell Scheduling and Negotiation Techniques for 6TiSCH Networks Under Bursty Traffic

**DOI:** 10.3390/s25051418

**Published:** 2025-02-26

**Authors:** Je-Hyeong Lee, Sang-Hwa Chung

**Affiliations:** Department of Information Convergence Engineering, Pusan National University, Busan 46241, Republic of Korea; dlwpgud9684@gmail.com

**Keywords:** 6TiSCH, minimal scheduling function, cell allocation, 6top protocol, wireless sensor network

## Abstract

6TiSCH networks adopt the IEEE 802.15.4e-based TSCH protocol to support efficient and reliable communication in low-power and lossy network (LLN) environments. However, under bursty traffic conditions, the traditional minimal scheduling function (MSF)-based scheduling technique cannot effectively handle the traffic load and suffers from packet queue overflow. In this study, we propose two main techniques to solve these problems. The first technique, dynamic cell cycle adjustment, dynamically adjusts the cell addition and deletion cycles based on the link quality and packet queue utilization to prevent packet queue overflow and efficiently use limited cell resources. The second technique, the parent node 6P transaction forwarding technique, is designed to pre-forward cell addition requests to higher nodes along the path when the cell utilization exceeds a set threshold due to traffic spikes at the lower nodes, so that the higher nodes can perform 6P negotiation immediately without waiting for MAX_NUMCELLS cycles. This minimizes the cell addition delay and prevents packet queue overflow. The simulation results show that the proposed technique has a high packet delivery ratio (PDR), low latency, and energy efficiency compared to conventional MSF, IMSF, and LMSF in various traffic environments. In particular, it maintains stable performance while preventing packet overflow under bursty traffic conditions. This work contributes to the optimization of scheduling and cell negotiation in 6TiSCH networks to improve the network efficiency and reliability in IoT environments.

## 1. Introduction

The Internet of Things (IoT) [1,2] is a technology that connects physical objects to a network to collect, process, and analyze data, and it is playing a pivotal role in many areas of modern society. IoT technologies are applied in many areas, including industry, smart cities, healthcare, and logistics management, and are helping to make human life more convenient and efficient. At the core of these technologies is the ability to increase the connectivity between physical devices and efficiently utilize the data they generate. In particular, the IoT enables data-driven decision-making, real-time situational monitoring, and predictive behavior.

The Industrial Internet of Things (IIoT) is one of the main applications of the IoT and is widely used in smart factories, automated production processes, industrial safety management, and more. In a factory environment, the IIoT plays an important role in maximizing productivity, enhancing safety, and reducing costs by connecting various machines and sensors to collect and analyze data. Particularly, the IIoT facilitates predictive maintenance by continuously monitoring machine conditions, preventing unexpected failures, and optimizing operational efficiency. However, to achieve this, highly reliable and low-latency communication is essential, as real-time data exchange is critical for automation and production control.

TSCH is an essential communication protocol in IoT and IIoT environments, designed to ensure reliability and reduce communication interference. TSCH minimizes the possibility of collisions by ensuring that each node transmits data only in predefined time slots, and it periodically changes frequencies to address interference and multipath fading. This feature is particularly crucial in industrial environments, where electromagnetic interference (EMI) from heavy machinery and signal reflection from metal structures can severely degrade wireless communication reliability. By employing time-synchronized channel hopping, TSCH significantly enhances the data transmission stability, making it highly suitable for industrial automation applications.

In addition to industrial automation, TSCH-based scheduling techniques can be applied in other critical domains, such as smart cities and healthcare. In smart city applications, low-power and highly reliable communication is essential for traffic monitoring, smart grid management, and real-time environmental sensing. Similarly, in healthcare environments, IoT devices used for remote patient monitoring, medical sensor networks, and emergency response systems require low-latency and energy-efficient data transmission mechanisms to ensure their reliability. Since these domains also demand highly predictable and adaptive communication scheduling, TSCH- and 6TiSCH-based techniques can play a crucial role in enhancing network performance across diverse IoT environments.

However, TSCH does not provide a specific way to create or manage communication schedules on its own. This means that additional design is required to optimize the utilization of network resources [3]. In a TSCH network, scheduling plays a key role in preventing data collisions and maintaining network performance. However, TSCH itself is limited in that it does not include specific algorithms or protocols to solve these scheduling problems, leaving it to the network operator or application designer to supplement it [4].

To address these issues, an IPv6-based TSCH mode (6TiSCH) architecture was developed. 6TiSCH adds a scheduling function (SF) for efficient management of network resources on top of the basic structure of TSCH and includes the 6P protocol to negotiate cell additions and deletions. Recent research has focused on optimizing 6TiSCH networks by enhancing the scheduling functions and improving the reliability under dynamic traffic conditions [5]. For instance, studies have explored hierarchical reinforcement learning-based TSCH schedulers for industrial IoT environments [6]. 6TiSCH supports the scalability and reliability of TSCH networks and has a flexible structure that can adapt to traffic changes. These features greatly enhance the performance of the network and help it work effectively in a variety of application scenarios.

The minimal scheduling function (MSF), a widely used scheduling algorithm in 6TiSCH, dynamically manages network resources by determining when to add and delete cells based on the cell utilization [7]. However, the MSF has the following limitations in environments with increasing traffic. First, the MSF does not fully account for the link quality (ETX) or traffic load because it only determines the cell addition and deletion based on the cell utilization. Second, the MSF waits a fixed number of MAX_NUMCELLS cycles before deciding whether to add or delete cells, which limits the network’s ability to add cells in a timely manner in situations of rapid traffic growth. This can lead to packet queue overflow issues.

Therefore, a new approach is needed to address the traffic volatility problem and increase the reliability of 6TiSCH networks. This paper proposes two main techniques to address this issue. The first technique is to pre-forward 6P negotiation to higher nodes to expedite 6P negotiation-based cell addition under increased traffic [8]. This technique is designed to reduce the negotiation latency and allow higher nodes to immediately perform cell addition negotiation even under an increased traffic load. The second technique is to dynamically adjust the cell addition and deletion cycles based on the link quality and transmission queues. This allows the network performance to remain stable even as the traffic fluctuates. In addition to proposing these techniques, extensive simulations are conducted using the 6TiSCH simulator to evaluate their performance under various traffic conditions, including bursty traffic scenarios. The experiments measure key performance indicators, such as the packet delivery ratio (PDR), end-to-end latency, and energy consumption, to validate the effectiveness of the proposed approach. Furthermore, a comparative analysis with existing scheduling functions, including the MSF, LMSF, and IMSF, is performed to demonstrate the efficiency and scalability of the proposed techniques in different network configurations. The results confirm that the proposed techniques significantly improve the network reliability and adaptability to dynamic traffic patterns.

- Dynamically adjusts the cell add and drop cycles based on the ETX and transmission queue to reduce the packet overflow in the network and reduce unnecessary resource occupation.
- The proposed 6P negotiation pre-forwarding technique is designed to quickly perform cell additions without waiting for MAX_NUMCELLS cycles by pre-forwarding the transaction to higher nodes along the path when the traffic spikes at lower nodes. This reduces the cell addition latency and prevents packet queue overflow.

This paper is organized as follows. Section 2 covers the related work, providing a comprehensive overview of prior research relevant to this paper. Section 3 delves into the background theory, explaining the fundamental concepts of the 6TiSCH [9] network stack, TSCH [10], 6top, RPL, and MSF, which equips the reader with the foundational knowledge required to understand the proposed techniques. Section 4 discusses the limitations of the existing MSF schemes and introduces the design principles and operational mechanisms of the proposed 6P negotiation forwarding technique and the dynamic cell check cycle management technique aimed at addressing these limitations. Section 5 presents the experimental verification of the proposed techniques using the 6TiSCH simulator and demonstrates their superiority by comparing their performance with existing MSF schemes. Section 6 provides a detailed scalability analysis, discussing how the proposed approach performs as the network scales to 100+ nodes and evaluating its computational feasibility in large-scale deployments. Additionally, a dedicated limitations section is included to outline the potential drawbacks of the proposed techniques, such as the computational overhead in real-time environments, adaptability to diverse network conditions, and trade-offs between performance and complexity. Finally, Section 7 concludes the paper, summarizing the findings and contributions of this research.

## 2. Related Work

Several approaches have been studied to improve the cell assignment performance in 6TiSCH networks. Representative of the existing work are the lightweight minimal scheduling function (LMSF) [11] and the improved minimal scheduling function (IMSF) [12]. Both are designed to improve on the MSF’s limitations and add cells dynamically. However, these approaches still exhibit several limitations. Please see the details from Table 1.

First, the LMSF relies on a Poisson distribution for traffic prediction, which may not be accurate in environments with highly dynamic and unpredictable traffic variations. If the prediction is incorrect, the LMSF may fail to allocate enough cells, leading to packet queue overflow and increased latency. Conversely, overestimation of the required cells may result in unnecessary resource occupation.

Second, the IMSF introduces a more adaptive allocation mechanism by considering both the expected cell utilization and ETX values. However, its batch allocation method can cause over-provisioning, leading to inefficient use of the available resources, particularly when traffic conditions fluctuate unpredictably.

Third, both the LMSF and IMSF do not proactively handle sudden increases in the traffic load. They react only after a threshold is exceeded (e.g., 75% utilization), which may delay the addition of necessary cells and cause congestion. This reactive approach limits their ability to quickly accommodate bursty traffic scenarios. Finally, the MSF methods do not explicitly consider the network state when making cell allocation decisions. As a result, network congestion may occur during traffic surges, leading to excessive packet drops due to queue overflow.

To address these challenges, the proposed method introduces dynamic cell cycle adjustment based on real-time queue utilization and preemptive 6P transaction forwarding. By dynamically modifying the cell check period and proactively negotiating the cell assignments before congestion occurs, the proposed approach minimizes the packet loss and improves the network efficiency, particularly in bursty traffic conditions.

## 3. Background

### 3.1. TSCH

Time-slotted channel hopping (TSCH) is a medium access control (MAC) layer protocol defined in the IEEE 802.15.4e standard [13] that focuses on maximizing the network’s reliability and energy efficiency by efficiently utilizing the time and frequency resources. TSCH works based on the network’s time synchronization and is designed to ensure that data are only transmitted in predefined time slots. This prevents interference and collisions and ensures stable and reliable data transmission in various environments.

All the nodes synchronize their time in a network based on the same absolute slot number (ASN). The ASN starts at zero at network initialization and increments by one for each subsequent timeslot, ensuring all the nodes share the same time reference. TSCH organizes the time slots based on these ASNs, and within a slot frame, each slot is defined as transmitting (TX), receiving (RX), or idle. Nodes only communicate in the slots assigned to them, which allows for efficient, collision-free data transfer.

In TSCH (time-slotted channel hopping), a slot frame serves as the fundamental unit for scheduling communication resources in a network. Each slot is defined by a slot offset, which represents its position within the slot frame, and a channel offset, which determines the frequency channel used for communication. TSCH employs a frequency hopping mechanism that periodically changes the communication channel to minimize interference and collisions, ensuring more reliable data transmission. This process is governed by Equation (1), which determines the frequency channel a node will use in a given slot. In this equation, CH represents the channel index used for transmission and reception, while macHopSeq denotes the predefined hopping sequence assigned to the node. The absolute slot number (ASN) serves as a global counter that increments with each slot, and the channel offset, denoted as CHoffset, is an assigned value that influences the node’s frequency selection. The length of the hopping sequence array, denoted as macHopSeqLen, ensures that the frequency selection follows a cyclic pattern. By applying this equation, the nodes in a TSCH network dynamically select their communication frequencies, improving the network reliability and reducing external interference.

CH = macHopSeq [(ASN + CHoffset)% macHopSeqLen] (1)

**Figure 1:**
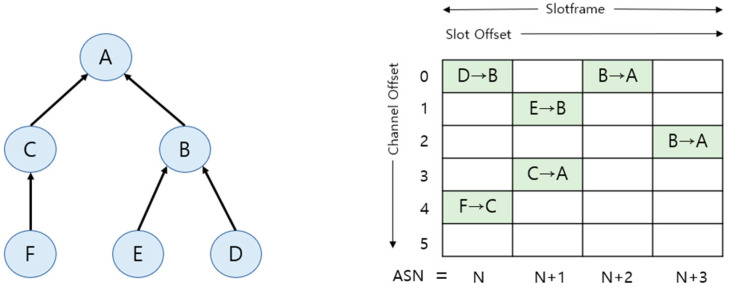
TSCH slot frame.

For example, the structure of a TSCH slot frame in Figure 1 visually describes the process of data transmission between nodes in a network. In the tree structure of Figure 1, parent nodes, such as A, B, and C, send and receive data to and from child nodes, such as D, E, and F, utilizing the slot offset and channel offset of the TSCH slot frame. Figure 1 shows that data transfers such as D → B, B → A, and E → B are accomplished by combining specific time slots and channel offsets. This design ensures reliable data transmission even in interference-prone environments and effectively overcomes wireless network challenges such as multipath fading through frequency dispersion techniques.

TSCH also uses keep-alive (KA) packets and enhanced beacon (EB) [14] messages to continuously maintain the time synchronization within the network. These messages enhance the reliability and stability of the communication between network nodes and are designed to maintain network performance, especially in high interference or complex multipath environments. For example, in factory environments where interference from metallic structures is common, TSCH can still transmit data reliably and efficiently with effective time synchronization.

### 3.2. 6P

The 6P protocol is an important mechanism designed to manage cell resources in the 6TiSCH network efficiently. The network utilizes the 6P protocol to dynamically add or delete cells based on traffic fluctuations. Cell utilization refers to the percentage of cells used by a node to send and receive data, and it is the basis for resource management in the network. If the number of used cells exceeds the upper limit (Lim_NumCellsUsed_High) or falls below the lower limit (Lim_NumCellsUsed_Low), 6P negotiation for adding or deleting cells is executed.

Moreover, 6P negotiation works by negotiating cell additions and deletions between nodes. For example, when a node determines that it needs to add cells, it sends a 6P ADD request message to request the number of cells it needs, a list of candidate cells (CellList), and cell options (CellOptions). The node receiving the request selects the cells from the CellList and responds with a 6P response message. The process is finally completed by exchanging an ACK message. This negotiation contributes to increasing the efficiency of network resources and reducing unnecessary cell resources. 

**Figure 2:**
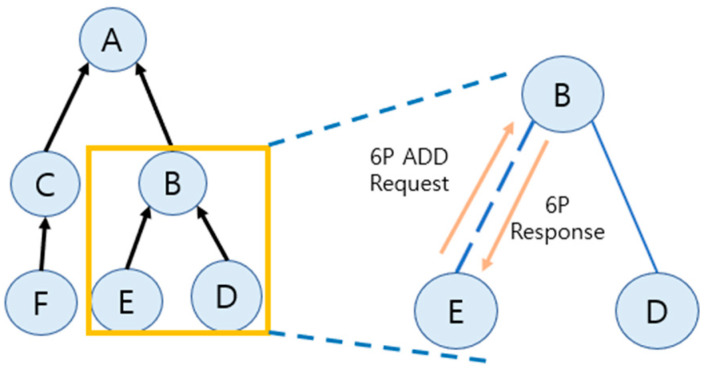
6P Negotiation request and response process.

Figure 2 visually represents how 6P negotiation is performed in a tree-based network. In the tree structure, node B receives a 6P ADD request from child node E, negotiates to select a suitable cell, and returns a response (6P response). This negotiation process plays an important role in resource management and maintaining the stability of the network.

The 6P protocol is also closely related to the time–frequency slot structure of TSCH. TSCH combines time slots and channel offsets to reduce network interference and ensure communication reliability. The frequency used by each node in the network is determined by the formula in Equation (1). For example, in a 6P negotiation between nodes E and B, the TSCH slot frames and channel offsets are combined to ensure that data are transmitted efficiently and without interference.

**Figure 3:**
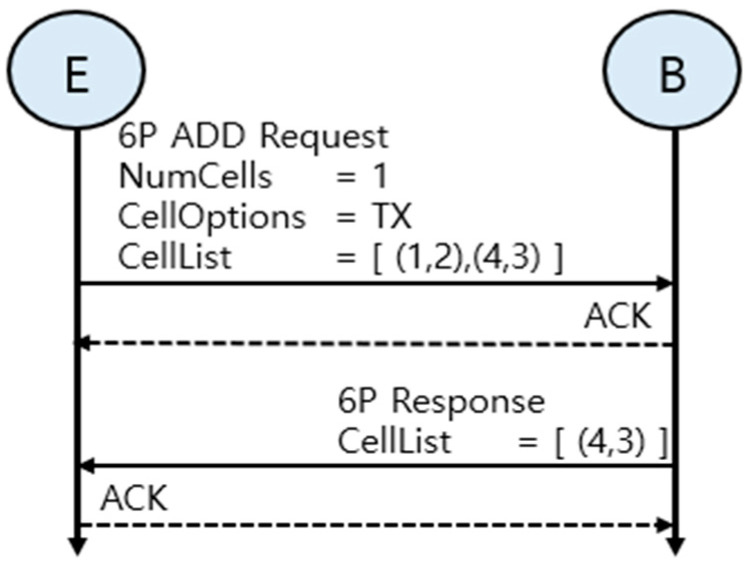
Example 6P 2-step transaction.

Figure 3 illustrates the 6P message exchange process in detail. Figure 3 visually illustrates the steps where node E requests additional cells via a 6P ADD request message, and node B responds by selecting a suitable cell based on a list of candidate cells. Finally, the negotiation is terminated with an ACK message. This message exchange enables efficient cell resource management in the 6TiSCH network.

The 6P (6top protocol) plays a role in dynamically managing cell resources in the 6TiSCH network, providing a mechanism to adapt to changes in network traffic. The protocol efficiently allocates cell resources through a negotiation process for cell addition and deletion and maintains the performance and stability of the network. The 6Ps share the status of specific cells through message exchanges between each node and then dynamically add necessary cells or remove unnecessary cells based on this. This approach reflects the dynamic nature of the network, reducing the waste of resources and improving communication efficiency.

### 3.3. RPL

The routing protocol for low-power and lossy networks (RPL) is designed to operate in low-power and high-loss environments and plays an important role in organizing and maintaining reliable data transmission paths in 6TiSCH networks [10,11]. The RPL organizes the network based on a directed acyclic graph (DAG), which forms a hierarchical tree structure centered on a root node. In this structure, the root node acts as the topmost node, and other nodes exchange data with the root node through its parent node. Each node on the network exchanges routing information via DIO (DODAG information object) messages and sends data via DAO (destination advertisement object) messages. This allows the network to adapt to dynamically changing traffic conditions flexibly [15].

In particular, the RPL uses the expected transmission count (ETX) value to evaluate the reliability of a route. The ETX represents the average number of transmissions required to successfully deliver a data packet to its destination over a particular path, with lower values indicating better path quality. For example, an ETX value of 1 indicates that the path is stable and reliable. In contrast, a value of 3 or higher indicates that the path is of low quality, increasing the likelihood of data loss, and should be rejected. This evaluation method is beneficial for maintaining high-quality routes within the network.

The working mechanism of the RPL is to propagate routing information from the root node to the child nodes, allowing each node to choose the best parent node for itself. In this process, the ETX value is used as one of the criteria for selecting a parent node, and nodes preferentially select routes with lower ETX values to maximize the data transfer efficiency. In addition, the ETX value is dynamically updated, allowing each node to choose the optimal path according to traffic changes in the network.

The ETX is a key evaluation metric for the RPL protocol and plays an important role in managing and selecting routes in the network. For example, when the traffic load on the network increases or the quality of a particular path degrades, the RPL selects an alternative path based on the ETX value to minimize packet loss and ensure the reliability of data transmission. User studies also utilize the ETX values provided by the RPL to dynamically adjust the cell addition and deletion cycles to enable efficient resource management in the face of traffic fluctuations.

### 3.4. MSF

The minimal scheduling function (MSF) is designed to efficiently manage cell scheduling in 6TiSCH networks. It dynamically adapts to changes in network traffic and aims to reduce the packet loss and minimize latency. The MSF works based on cell utilization and network conditions to reduce the wastage of cell resources and increase the communication reliability. The MSF uses three main types of cells to manage network resources: minimal cells, autonomous cells, and negotiated cells.

**Figure 4:**
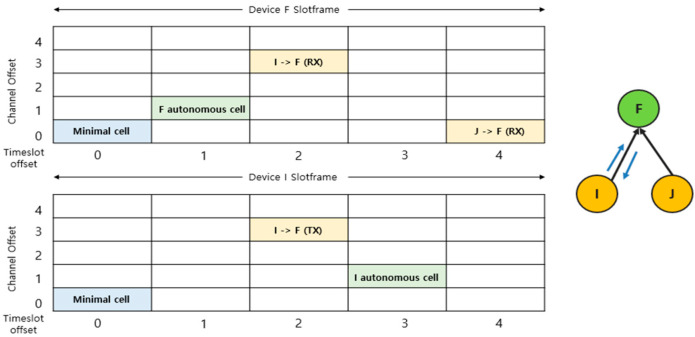
Slot frame with cells allocated using an MSF.

Minimal cells are reserved for basic data transfers between network nodes, synchronization, and essential control message exchange. Autonomous cells are cells that each node allocates and uses on its own. They are designed to maintain the primary communication path between nodes when the traffic changes. Negotiated cells are dynamically added or deleted through a negotiation process between nodes using the 6P (6top protocol) and are mainly used to optimize cell resources in traffic situations. Figure 4 shows a slot frame visualization of the cell scheduling characteristics of such an MSF. Data transmission and reception between nodes are based on a combination of specific time slots and channel offsets to reduce interference and ensure reliable communication.

The MSF works based on cell utilization (NumCellsUsed) and the elapsed time of cell usage (NumCellsElapsed). When the cell utilization exceeds the upper limit (LIM_NUMCELLSUSED_HIGH), it adds cells through 6P negotiation, and when it falls below the lower limit (LIM_NUMCELLSUSED_LOW), it deletes unnecessary cells. This characteristic provides a flexible response to network traffic changes and reduces unnecessary resource waste. 

Algorithm 1 shows how the MSF works, comparing the upper and lower bounds based on the number of cells used and the elapsed time to generate the necessary negotiation. This approach utilizes the 6Ps to adjust the cells and dynamically manage the network resources [16].
**Algorithm 1** MSF1:**Input:**2:
      **MAX_NUM_CELLS**
3:
      **NumCellsElapsed**
4:      NumCellsUsed5:
      **LIM_NUMCELLSUSED_HIGH**
6:
      **LIM_NUMCELLSUSED_LOW**
7:**Output:**8:      ADD/DEL negotiated cell9:**IF** NumCellsElapsed > MAX_NUM_CELLS **then**10:      **If** NumCellsUsed > LIM_NUMCELLSUSED_HIGH11:             6P to **ADD** one negotiated cell12:      **If** NumCellsUsed > LIM_NUMCELLSUSED_LOW13:             6P to **DEL** one negotiated cell

Cell negotiation in 6TiSCH networks plays an essential role in efficiently managing network resources and ensuring the reliability of data transmission. The cell negotiation process consists of a mechanism where network nodes cooperate to dynamically add or delete cells based on the traffic load. Generally, this negotiation is performed when the cell utilization meets certain conditions, which is achieved through the 6P negotiation.

Cell Utilization = (NumCellsUsed)/(NumCellsElapsed)(2)

**Figure 5:**
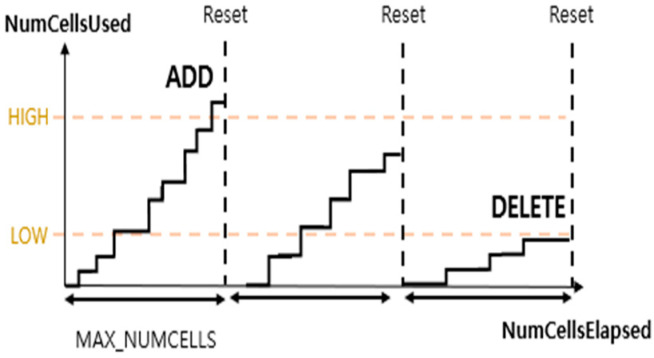
Principle of cell addition and deletion characteristics in an MSF.

The cell negotiation method starts by checking if the performance condition is met. The cell utilization is calculated using Equation (2). If the calculated value exceeds the upper limit (LIM_NUMCELLSUSED_HIGH) of 75% or falls below the lower limit (LIM_NUMCELLSUSED_LOW) of 25%, negotiation is initiated to maintain the efficiency of network resources. This is useful to prevent performance degradation due to overuse or underuse of network resources. If the upper limit is exceeded, it is determined that additional cells are needed, and an ADD request is made. If it falls below the lower limit, a DELETE request will be made to delete unnecessary cells. Figure 5 provides a visual representation of this cell utilization evolution and shows that after the MAX_NUMCELLS period, negotiation is performed if the cell utilization falls outside of the appropriate range.

Moreover, 6P negotiation implements cell addition or deletion through negotiation between the requesting and responding nodes. The requesting node sends an ADD request or DELETE request message with the required information to the responding node, which selects the appropriate cell and responds with a response message. Finally, the negotiation is completed with an ACK message. This method dynamically manages cell resources and enables stable data transmission even in environments where the traffic changes frequently.

The existing methods have the advantage of efficiently managing network resources through cell negotiation. The ability to dynamically adjust cells as traffic increases and decreases contributes to maintaining network stability. However, there are also some drawbacks. Because the default method uses a fixed MAX_NUMCELLS cycle in environments with high traffic volatility, it does not respond well to rapid traffic changes. In particular, the structure of waiting for a fixed number of cycles and then performing cell negotiation can lead to packet queue overflow in situations where the traffic load suddenly increases.

## 4. Proposed Methodology for Cell Scheduling and Transaction Optimization

We propose two techniques to solve the existing problems and minimize the impact of packet overflow and the long occupation of limited resources. The first is the “dynamic cell cycle adjustment technique”, which dynamically adjusts the cell addition and deletion cycles. The second is the “6P transaction forwarding technique”, which proactively forwards the 6P negotiation in situations where the traffic increases.

### 4.1. Dynamic Cell Cycle Control Technique

**Figure 6:**
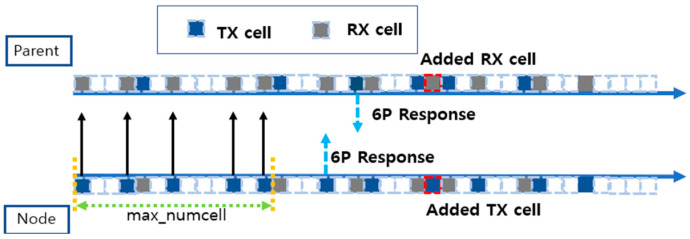
Cell addition process in a traditional MSF.

The traditional MSF method efficiently manages network cell resources by evaluating the cell utilization to determine the cell addition and deletion. To understand this, consider Figure 6, which is a visual representation of the cell utilization process and the relationship between nodes in the network and their parent nodes. Each square box represents a cell, with blue boxes representing transmit (TX) cells and gray boxes representing receive (RX) cells. Empty boxes are cells that have not yet been allocated and can be utilized for additional traffic.

At the top of Figure 6 is the parent node (Parent), and at the bottom is the child node (Node), which sends and receives data to and from the parent node to evaluate cell utilization. MAX_NUMCELLS is a check period for evaluating the cell utilization, which is used to observe the cell usage, NumCellsUsed and NumCellsElapsed, over some time and make cell addition and deletion decisions based on that. For example, if MAX_NUMCELLS is set to 100, the status of the cells used during the MAX_NUMCELLS period will be evaluated after 100 timeslots have passed.

NumCellUsed represents the number of cells used during the period for which the cell utilization is checked, and NumCellElapsed is the total number of cells allocated during that period. The ratio of these two values, Equation (2), represents the cell utilization and is used to decide whether to add or delete cells. When the cell utilization exceeds the upward threshold of 75%, the child node sends a cell addition request to the parent node via the 6P protocol. Conversely, if the cell utilization reaches the downward threshold of 25% or less, it requests to delete unnecessary cells.

The main limitation of existing techniques is that the cell utilization used to determine the cell addition and deletion is evaluated only after a fixed period, MAX_NUMCELLS. This can lead to over-provisioning, where cells are not added promptly in the face of network state changes, rapidly increasing traffic loads, preventing other nodes from using the required cell resources, or over-allocating cells. In particular, if the network quality is degraded, such as increasing the link quality estimation (ETX) values and transmit queue occupancy, the method of waiting for a fixed number of MAX_NUMCELLS cycles before deciding to add cells may delay the adding of needed cells, resulting in packet queue overflow. In contrast, instead of waiting for a fixed MAX_NUMCELLS period, it dynamically determines the optimal period based on the link quality and queue utilization, ensuring that necessary cell adjustments are performed proactively.

(3) $$LSF=\frac{ETX-1}{2}(1-min)$$

(4) $$QUF=1-\frac{Number\;of\;Packets\;in\;the\;queue}{Max\;queue\;size}(1-min)$$

The proposed dynamic cell period adjustment technique adaptively adjusts the MAX_NUMCELLS period based on the link state factor (LSF) and queue utilization factor (QUF) to address the inefficiencies in cell allocation. The minimum period factor (min) is introduced to prevent excessive short-term fluctuations in the cell allocation. The value of the min is set to 0.1, ensuring that the minimum cell check period remains at 10 slots. This prevents frequent and unnecessary cell reallocation, which could lead to inefficient resource utilization. The link state factor (LSF) is derived from the expected transmission count (ETX) metric, as defined in Equation (3), which quantifies the probability of successful data transmission between nodes. A lower ETX value (closer to 1) indicates better link quality, while a higher value (closer to 3) signifies a degraded link. The LSF is scaled within the range [0, 1-min], ensuring that better link conditions contribute to more stable cell allocations. The queue utilization factor (QUF), defined in Equation (4), represents the availability of queue resources within a node. The QUF value ranges from 0 to 1 min, where a value close to 1 indicates that the queue is mostly empty (high availability), while a value near 0 suggests that the queue is full (low availability). This ensures that the scheduling mechanism dynamically adapts the cell allocation period in response to varying queue loads, preventing network congestion while optimizing resource usage.

These factors are utilized to dynamically adjust the cell addition and deletion cycle using the following formula.

Dynamic cell check cycle = MaxNumCells × [(α∙LSF) + (β∙QUF) + min](5)

Unlike traditional MSF-based approaches, which rely on periodic scheduling checks at fixed intervals, our proposed technique dynamically adjusts the scheduling period in real time. This approach reduces unnecessary computations since it does not require maintaining a separate scheduling table or executing frequent scheduling updates at static intervals. Instead, the computational overhead is limited to lightweight calculations involving the LSF and QUF, both of which are derived from already available network metrics. In Equation (5), min sets the minimum value of the cell allocation cycle, which is important for managing the cell allocation and deletion cycle. Suppose the cell allocation cycle is too short. In that case, the cell additions and deletions may become excessively frequent to reflect only short-term network state changes, leading to problems such as over-provisioning or packet queue overflow due to cell shortage.

We experimentally analyzed the impact of adjusting the dynamic cell check period by setting the α and β values to set the weights of the LSF and QUF. The experiments compared the packet delivery ratio and average latency by setting α values to 0.1, 0.2, 0.3, 0.4, 0.5, 0.6, 0.7, 0.8, and 0.9 and β values to 0.9, 0.8, 0.7, 0.6, 0.5, 0.4, 0.3, 0.2, and 0.1 under the same conditions. The experiments used ten MOTEs and 1200 slot frames, including a normal interval of sending one packet every 10 s and a bursty traffic interval of sending one packet every 0.6 s between 800 and 1000 slot frames. The results showed that α = 0.5 and β = 0.5 gave the best results, with the highest PDR and lowest latency.

**Figure 7:**
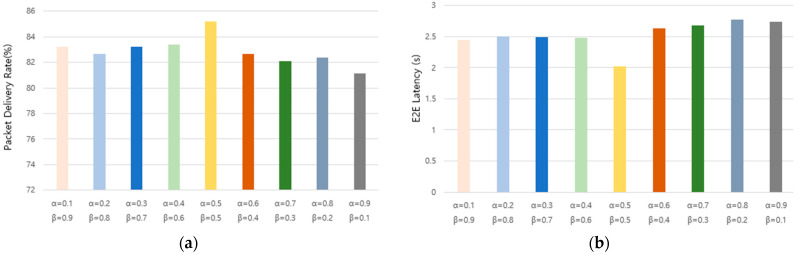
Packet delivery ratio and latency as a function of the α, β values. (**a**) Packet delivery ratio as a function of the α, β values. (**b**) E2E latency as a function of the α, β values.

Figure 7 presents a visual representation of the experimental results, demonstrating the impact of different α and β values on the network performance. The results indicate that setting α to 0.5 and β to 0.5 consistently yields the highest packet delivery ratio (PDR) while maintaining the lowest end-to-end (E2E) latency. To further refine the parameter selection, we conducted additional experiments by varying the α and β values with the following combinations: α = 0.1, β = 0.9; α = 0.2, β = 0.8; α = 0.3, β = 0.7; α = 0.4, β = 0.6; and α = 0.5, β = 0.5. These specific combinations were selected based on their relatively strong performance in the preliminary weighted experiments. The experiments were conducted in a simulated environment with 10 MOTEs over 1200 slot frames, incorporating both regular and burst traffic scenarios. The regular traffic interval consisted of one packet transmission every 10 s, whereas bursty traffic was introduced between slot frames 800 and 1000, where one packet was transmitted every 0.6 s. Additionally, we analyzed the effect of varying the minimum dynamic cell check period (min) by setting its value to 0.1, 0.2, and 0.3. The objective was to observe its impact on the packet delivery ratio (PDR) and average E2E latency.

**Figure 8:**
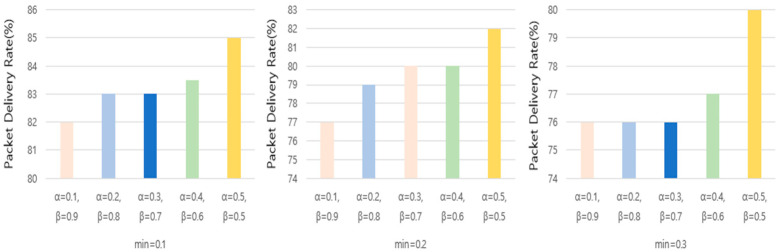
Packet delivery ratio with minimum dynamic allocation period.

**Figure 9:**
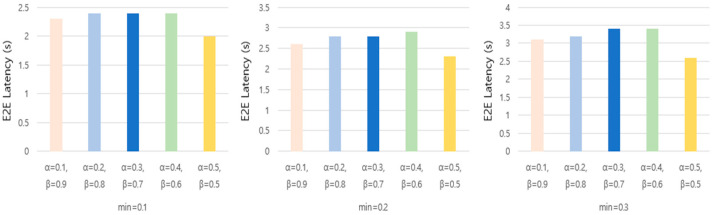
E2E latency with minimum dynamic allocation period.

Figure 8 illustrates the relationship between the PDR and different minimum period values, while Figure 9 presents the average E2E latency under the same conditions. Our findings indicate that different min values lead to variations in both the packet delivery rate and the latency. Specifically, when min was set to 0.1, the PDR remained relatively high across most α and β combinations, and the average latency was lower compared to the other settings. This suggests that reducing the minimum dynamic allocation period enhances the network responsiveness to traffic fluctuations while maintaining reliable packet delivery.

Algorithm 2 describes the dynamic cell check cycle algorithm, which optimizes cell scheduling in 6TiSCH networks by dynamically adjusting the cell addition and deletion cycles based on the network conditions. The algorithm operates by considering two key factors: link quality (LSF) and queue vacancy (QUF), which collectively determine the appropriate adjustments for the cell allocation. First, the algorithm calculates the link quality using the CALCULATE_LINK_QUALITY function, which retrieves the expected transmission count (ETX) value of the preferred parent node. A lower ETX value indicates a better-quality link. If the ETX value is 1, the LSF is set to 0, representing optimal link health. Otherwise, the LSF value is computed using Equation (3), normalizing it within the range [0, 1] to represent network stability numerically. Next, the algorithm assesses the queue vacancy state. It retrieves the current queue length via the GET_TX_QUEUE_LENGTH function and computes the QUF using Equation (4). A QUF value close to 0 suggests a high risk of queue overflow, whereas a value near 1 indicates sufficient queue availability. Using the computed LSF and QUF, the algorithm determines the adaptive cell allocation ratio using Equation (5). This ratio dynamically adjusts the cell addition and deletion cycles, balancing the link quality and queue status. Here, θ is a calibration constant that defines the minimum and maximum allocation cycles, set to 0.1 based on experimental results optimizing the PDR and latency. Finally, the adaptive_add_cell_ratio and adaptive_delete_cell_ratio values are updated through the UPDATE_CELL_UTILIZATION_RATIOS function, ensuring real-time adaptation to changing network conditions. When the network conditions deteriorate (high LSF and low QUF), the algorithm shortens the cell addition cycle to prevent packet overflow. Conversely, when the conditions improve (low LSF and high QUF), it increases the cycle to prevent over-provisioning. Similarly, for cell deletion, in a stable network (low LSF and high QUF), the deletion cycle is shortened to quickly free unused resources, whereas in unstable conditions (high LSF and low QUF), the cycle is extended to avoid premature cell removal and minimize packet loss.
**Algorithm 2** Dynamic 6P cell allocation adjustment1:**function** CALCULATE_LINK_QUALITY(mote, preferred_parent)2:      **return** mote.tsch.calculate_link_quality(preferred_parent)3:**end function**4:
5:**function** GET_TX_QUEUE_LENGTH(mote)6:      **return** mote.tsch.get_tx_queue_length()7:**end function**8:
9:**function** ADJUST_CELL_ALLOCATION_RATIO(mote, cell, sent_packet, tx_queue_len)10:      etx_value = mote.rpl.get_etx(preferred_parent)11:      min = 0.112:     if etx_value ≤1:13:            LSF = 014:     else15:            LSF = ((etx_value-1)/2)(1-min)16:      QUF = (1-(tx_queue_len/(self.mote.tsch.txQueueSize))(1-min)17:      alpha = 0.518:      beta = 0.519:
20:      adaptive_delete_cell_ratio = ((alpha * LSF) + (beta * QUF) + min)21:      adaptive_delete_cell_ratio = ((alpha * LSF) + (beta * QUF) + min)22:         UPDATE_CELL_UTILIZATION_RATIOS(mote, cell, sent_packet, adaptive_add_cell_ratio, adaptive_delete_cell_ratio)23:
      **end if**
24:**end function**

**Figure 10:**
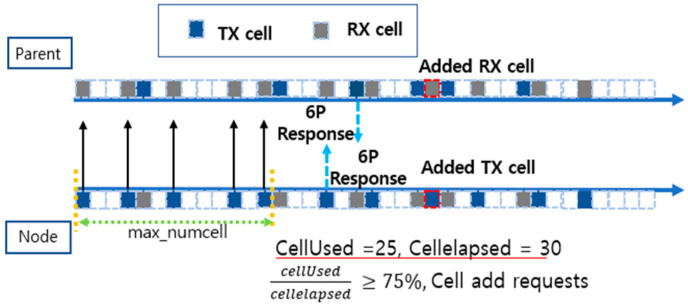
Cell addition process with dynamic cell cycle throttling.

In Figure 10, each square box represents a cell, with the blue boxes representing transmit (TX) cells, the gray boxes representing receive (RX) cells, and the empty boxes representing cells that have not yet been assigned. At the top is the parent node (Parent), and at the bottom is the child node (Node), which sends and receives data to and from the parent node, evaluates the network conditions, and decides to request additional cells based on the cell utilization.

For example, in Figure 6, MAX_NUMCELLS is initially set to 100. However, as a result of dynamically adjusting the cell allocation by calculating the network conditions, we can see in Figure 10 that the MAX_NUMCELLS cycle has decreased to 30. This reduces the frequency of adding cells when the network traffic spikes or the link quality deteriorates. NumCellUsed is measured to be 25 on the child node, NumCellElapsed is 30, and the cell utilization is 0.83 according to Equation (2), which exceeds 75%. Therefore, it sent a request for more cells to the parent node through the 6P protocol, and the parent node responded and allocated new cells. In the proposed technique, MAX_NUMCELLS is dynamically adjusted to allow the child nodes to fulfill cell addition requests faster. This shows that as the network condition deteriorates, more cells can be added quickly to prevent packet overflow and maintain network stability.

### 4.2. Forwarding 6P Transactions

In a 6TiSCH network, when a terminal node’s traffic increases, the traffic is forwarded along the path to the parent node and then to the root node, resulting in an increase in cell utilization at each hop along the path. In this situation, each node along the path will perform additional cell allocation requests through 6P negotiation to handle the traffic forwarded from the terminal node.

**Figure 11:**
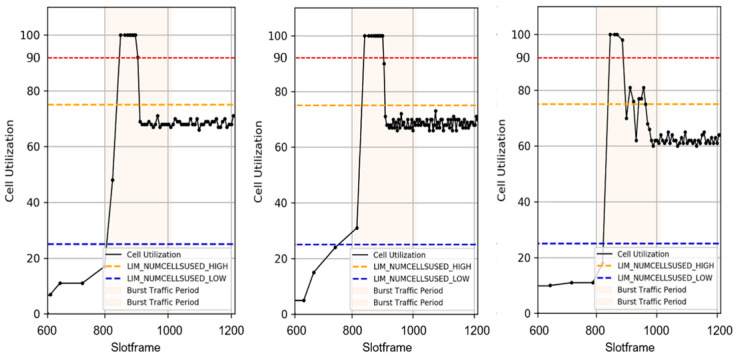
Changes in the cell utilization along the path as the traffic changes.

To understand this more concretely, Figure 11 visually represents how the cell utilization changes at each hop along the path when the traffic increases at the end nodes. In Figure 11, the x-axis of each graph represents the slot frame time, which represents the passage of time while the network is operating. The y-axis represents the cell utilization, a quantitative representation of the percentage of cells each node uses. The graph on the left shows the evolution of the cell utilization at the first hop, where the cell utilization is initially low. However, it then rises sharply as the traffic increases at the end nodes. The middle graph shows the change in the cell utilization at the second hop, which shows a further increase in the cell utilization due to the traffic forwarded from the first hop. Finally, the right graph shows the cell utilization at the last hop close to the root node, visualizing the gradual increase in the cell utilization as traffic is forwarded to higher nodes along the path. These results show that the increase in traffic at the terminal nodes causes a concomitant increase in the cell utilization at each hop along the path. The 6P transaction forwarding for cell addition during traffic growth is a process that allows the parent node to quickly detect and respond to the rapid increase in cell utilization at the lower nodes. This allows the parent node to perform cell additions before reaching cell saturation, avoiding packet queue overflow and delays.

**Figure 12:**
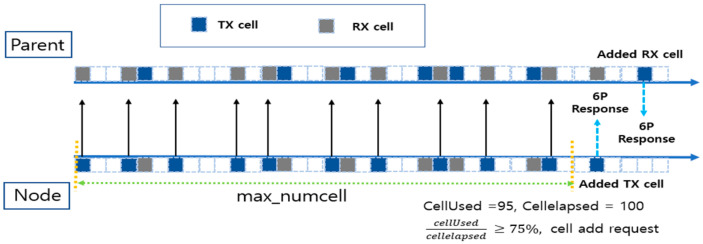
Cell negotiation process with max_numcell in a traditional MSF.

In a 6TiSCH network, when a terminal node’s traffic increases, it is forwarded to its parent node, and each node performs a cell addition request through 6P negotiation to handle it. However, in the traditional approach, the cell utilization must be calculated after the MAX_NUMCELLS period to make a cell addition request, which causes a delay, resulting in packet overflow. Figure 12 visually illustrates the problem with this traditional approach.

In Figure 12, each square box represents a cell, with the blue boxes representing transmit (TX) cells, the gray boxes representing receive (RX) cells, and the empty boxes representing cells that are not yet allocated. “Parent” at the top of the figure represents the parent node, and “Node” at the bottom represents the child node. The child node sends and receives data to and from the parent node, waits for MAX_NUMCELLS cycles, calculates the cell utilization, and adds cells.

However, in the process, a request to add cells is not made until MAX_NUMCELLS cycles have elapsed, so it is impossible to add cells quickly when the traffic increases rapidly at the child node. In particular, even if the traffic increases rapidly at the child node, the parent node waits for MAX_NUMCELLS cycles to calculate the cell utilization and add cells, increasing the possibility of packet overflow at the parent node. Also, even if a request to add cells is made, the top nodes may take a long time to allocate cells before reaching the stable region of 25% to 75%. If the initial number of cells is low, getting enough cells to handle the traffic load reliably can take a long time. This delay increases the likelihood of packet overflow due to high traffic before the network stabilizes [17].

The proposed technique is designed to overcome the limitations of the MSF, the existing cell scheduling technique in 6TiSCH, by reducing the delay during the MAX_NUMCELLS cycle and preventing packet overflow. The MSF determines whether to add or delete cells by calculating the cell utilization after the MAX_NUMCELLS cycle. However, in scenarios where the traffic increases rapidly at a lower node, the cell utilization of the parent nodes along the path also increases. In such cases, the parent nodes are unable to add cells until the MAX_NUMCELLS cycle has elapsed, which increases the likelihood of packet overflow due to accumulated traffic. To address this issue, Algorithm 3 presents the 6P transaction trigger mechanism, which proactively propagates the 6P negotiation request along the routing path. The process begins when a child node detects that its queue load exceeds a predefined threshold (90%). At this point, the node initiates a 6P transaction request to its preferred parent node using the TRIGGER_6P_TRANSACTION(node, threshold) function. This function first retrieves the preferred parent of the node. If no parent exists, the function terminates immediately. Otherwise, it evaluates whether the queue load exceeds the given threshold. If the queue load is above the threshold, a SEND_6P_REQUEST(node, parent) message is sent to the parent, and the TRIGGER_NEXT_PARENT(parent, threshold) function is called to propagate the request further. The TRIGGER_NEXT_PARENT(currentNode, threshold) function ensures that the negotiation request is forwarded up the routing path when necessary. This function iterates through the parent nodes along the path by retrieving each node’s preferred parent. If a parent node’s queue load is below the threshold or if no further parent exists, the process stops by invoking STOP_TRANSACTION(currentNode). Otherwise, the function forwards a SEND_6P_REQUEST(currentNode, nextParent) message to the next parent and continues the process iteratively. This cascading 6P negotiation mechanism ensures that the parent nodes allocate additional cells in advance before network congestion worsens, rather than waiting for the MAX_NUMCELLS cycle to complete. By dynamically adjusting the cell allocations through this proactive negotiation strategy, the proposed approach minimizes the packet drops due to queue overflow and significantly improves the end-to-end latency. The detailed steps of this process are outlined in Algorithm 3.
**Algorithm 3** The 6P transaction trigger1:**function TRIGGER_6P_TRANSACTION(node, threshold)**2:      parent = node.getPreferredParent()3:      **if** parent == None then4:
        **return**
5:
      **End if**
6:
7:      **if** node.queueLoad > threshold then8:          SEND_6P_REQUEST(node, parent)9:          TRIGGER_NEXT_PARENT(parent, threshold)10:
      **else**
11:          STOP_TRANSACTION(node)12:        end if 13:**end function**14:**function** TRIGGER_NEXT_PARENT(currentNode, threshold)15:  while currentNode ! = None do16:     nextParent = currentNode.getPreferredParent()17:     **if** nextParent == None or currentNode.queueLoad ≤ threshold then18:      STOP_TRANSACTION(currentNode)19:      break20:**else**21:    SEND_6P_REQUEST(currentNode, nextParent)22:    **end** if23:    currentNode = nextParent24:  **end** while25:    **end** function

**Figure 13:**
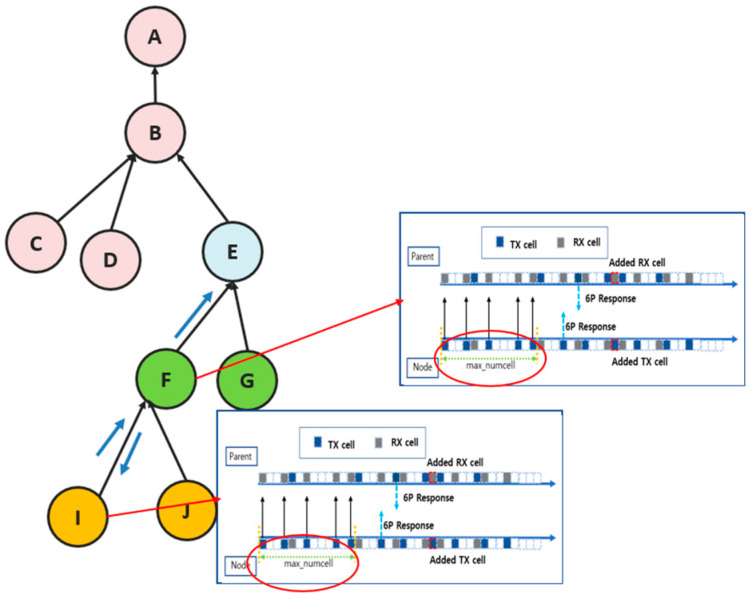
Traditional 6P transaction handling.

**Figure 14:**
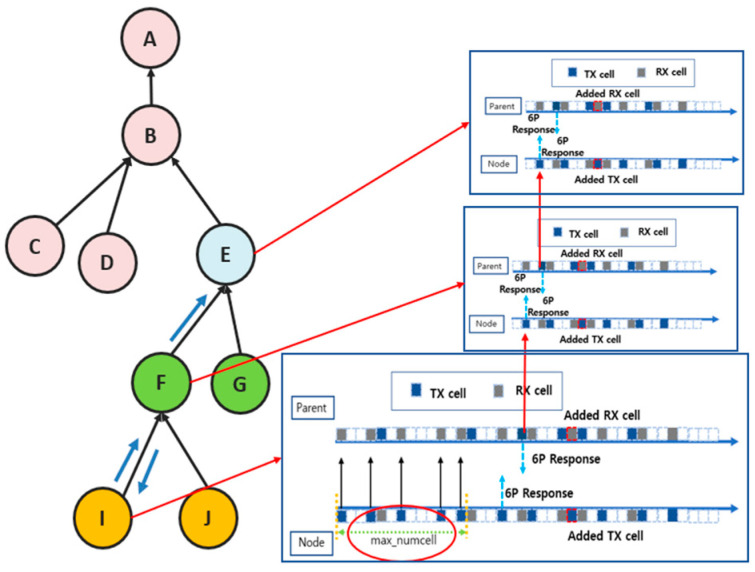
Proposed 6P transaction forwarding scheme.

Figure 13 and Figure 14 visually compare the existing and proposed techniques, respectively. In the traditional technique, even if a cell addition request occurs due to traffic growth at a lower node, the upper node waits a fixed number of MAX_NUMCELLS cycles to calculate the cell utilization before performing the cell addition. This approach has the problem that cells cannot be added quickly under heavy traffic because all the nodes along the path must wait for MAX_NUMCELLS sequentially. As a result, the cell utilization takes a long time to reach the safe zone (25% to 75%), which increases the likelihood of problems such as packet overflow.

Figure 13 shows the situation when node I experiences increased traffic and requests to add a cell. Node I can wait for MAX_NUMCELLS cycles to elapse before sending the 6P request to its parent node F to calculate the cell utilization. Parent node F also calculates the cell utilization after the same MAX_NUMCELLS cycle and processes the 6P request. This process is repeated at each node along the path until the root node A is reached. This is why there is a delay in the cell addition time under traffic growth. In particular, as the traffic growth at lower nodes propagates to higher nodes, the cell utilization takes a long time to reach a stable range (25% to 75%). As a result, the network performance degrades, and the probability of packet overflows increases significantly.

On the other hand, Figure 14 shows the situation with the proposed technique. In the proposed technique, when the cell utilization exceeds a threshold of 90% due to increased traffic at a lower node, a 6P negotiation request is immediately forwarded to the higher node along the path. The parent node calculates the cell utilization without waiting for MAX_NUMCELLS cycles and quickly decides whether to add a cell. If the cell utilization exceeds the upper threshold, the parent node that received the 6P negotiation performs the cell addition and immediately forwards the 6P negotiation request to the next parent node. If the cell utilization of the parent node that received the cell negotiation is below the threshold value or the root node is reached, further 6P negotiation forwarding is stopped. Thus, the proposed technique reduces the delay of 6P negotiation compared to the existing techniques and quickly adjusts the cell utilization along the path to a stable range, effectively avoiding the packet overflow problem.

## 5. Performance Evaluation

In this study, experiments were conducted using the 6TiSCH simulator [18] to evaluate the performance of the proposed method. The number of nodes in the experimental environment was set to 10, 20, 30, and 40, and the experiments were repeated 10 times for each configuration to obtain the average results. Each run consisted of 1200 slot frames, with the length of a slot frame set to 101. The network uses the OF0-based RPL protocol, the TSCH network utilizes 16 channels, and the timeslot length is 10 ms. In addition, the TSCH transmission queue size was set to 10 to assume a limited resource environment. Table 2 displays the parameter simulations along with our proposed approach.

To evaluate the impact of bursty traffic, the periods were set to 0.8, 1.0, 2.0, and 2.5 s, and the bursty traffic was configured to occur between 800 and 1000 slot frames. The scheduling algorithm uses the MSF to compare the existing and proposed techniques, and the distribution of the nodes is randomized.

### Performance Comparison Analysis

In this study, we measured the packet delivery ratio (PDR) and latency to compare the performance of the proposed technique with the existing MSF, LMSF, and IMSF. The experimental environment was conducted with 10, 20, 30, and 40 motes operating on a random topology with bursty traffic (0.8). Please see the details from Table 3 and Table 4.

**Figure 15:**
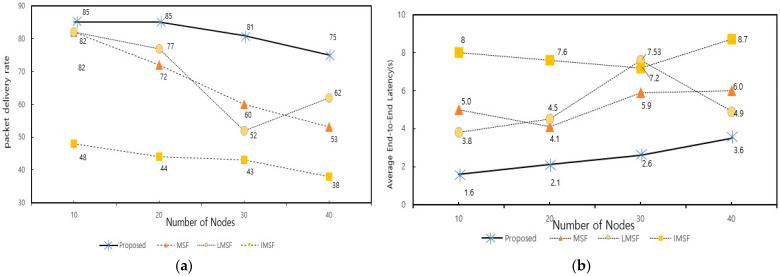
Comparison for different numbers of nodes under bursty traffic. (**a**) Comparison of the PDR by the number of nodes. (**b**) Average E2E latency by the number of nodes.

Figure 15 shows the comparison of the packet delivery ratio (PDR) and average latency of the proposed technique, conventional MSF, LMSF, and IMSF, respectively, as the number of nodes increases. The proposed method was tested in a bursty traffic environment for each node count (10, 20, 30, 40) with a packet generation period of 0.8 s. Based on this, the experiments were conducted, and the proposed method recorded the highest PDR and lowest latency.

**Figure 16:**
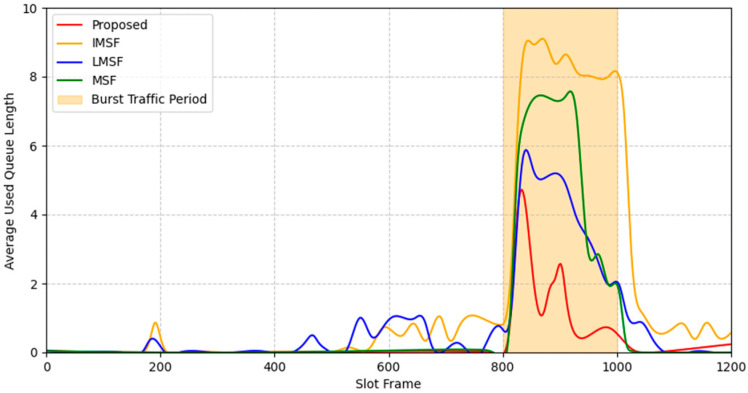
Average queue length over slot frames under bursty traffic.

The high PDR achieved by the proposed method can be attributed to its efficient queue management, which prevents packet drops due to queue overflow. To further illustrate this, Figure 16 presents the average queue length over the slot frames for each scheduling technique under bursty traffic conditions. For each configuration, the packet generation was set to 0.8 s, the maximum queue size was set to 10 packets and each experiment was repeated five times to obtain an average value. The results show that the conventional MSF, LMSF, and IMSF experience significant queue buildup during high-traffic periods (highlighted section), leading to packet loss when the queue exceeds its capacity. In contrast, the proposed method dynamically adjusts the cell allocation cycle based on the packet queue occupancy, effectively mitigating queue overflow and ensuring smooth packet transmission.

In particular, when the number of nodes is 40, the PDR of the proposed method is 75%, which is about 40% higher than the existing MSF, and the average latency is reduced by about 40% to 3.5 s. This improvement is closely linked to the ability of the proposed method to prevent queue overflow, which allows efficient utilization of the available cells and reduces congestion-related delays. The LMSF and IMSF, on the other hand, showed relatively low PDR and high latency. The IMSF suffered from poor prediction accuracy due to rapid changes in the queue occupancy, resulting in excessive cell allocation and additional delays. The LMSF suffered from low PDR due to its window-based cell prediction, which could not respond quickly enough to traffic fluctuations. The traditional MSF did not respond adequately to traffic spikes due to its fixed-cycle computation delay, which led to packet drops when the queue exceeded its capacity.

**Figure 17:**
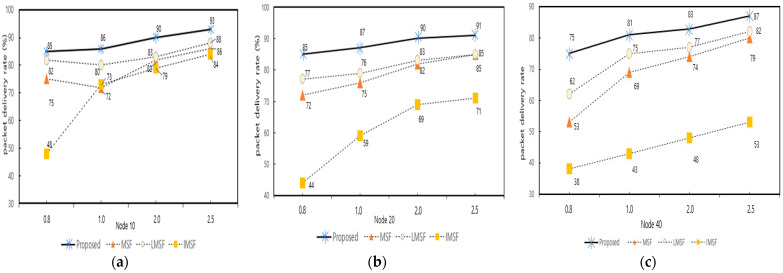
Packet delivery rate by node changes under bursty traffic. (**a**) PDR with bursty traffic variation on 10 nodes; (**b**) PDR with burst traffic variation on 20 nodes; and (**c**) PDR with bursty traffic variation on 40 nodes.

Figure 17 shows the comparison of the packet delivery ratio (PDR) of the proposed technique, traditional MSF, LMSF, and IMSF for different packet generation cycles (0.8, 1.0, 2.0, and 2.5) with bursty traffic when the number of nodes is 10, 20, and 40, respectively. The proposed method achieves the highest PDR across all the bursty traffic cycles and several nodes and is robust to changes in the network environment. In particular, as the number of nodes increases, the PDR performance of the conventional MSF, LMSF, and IMSF decreases significantly. At the same time, the proposed technique maintains a relatively high performance with a relatively moderate decrease in the PDR. For example, in Figure 17, when the number of nodes is 40 and the bursty traffic period is 0.8 days, the proposed method achieves a PDR of 75%, which is about 22%, 13%, and 37% higher than that of the traditional MSF (53%), LMSF (62%), and IMSF (38%), respectively. These results are attributed to the fact that the proposed method responds quickly to traffic changes in the network through the 6P transaction forwarding technique and minimizes the delay caused by the dynamic adjustment of cell add and delete checks. On the other hand, the IMSF recorded a low PDR due to the prediction errors caused by the queue occupancy changes, and the LMSF could not adapt quickly to traffic growth due to the fixed window-based prediction.

**Figure 18:**
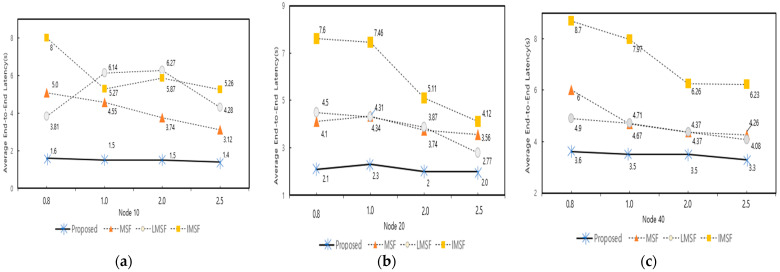
Average E2E latency by mode changes under bursty traffic. (**a**) Average E2E latency with burst traffic variation on 10 nodes; (**b**) average E2E latency with bursty traffic variation on 20 nodes; and (**c**) average E2E latency with bursty traffic variation on 40 nodes.

Figure 18 shows the average delay variation of the proposed technique, traditional MSF, LMSF, and IMSF with different bursty traffic ratios (0.8, 1.0, 2.0, and 2.5) at 10, 20, and 40 nodes, respectively. The proposed method achieves the lowest average latency for all the traffic conditions and several nodes. In particular, when the number of nodes is 10, and the bursty traffic ratio is 0.8, the proposed method shows a latency reduction of about 3.4 s compared to the traditional MSF. On the other hand, the LMSF and IMSF exhibited high latency. The MSF and LMSF predict cell additions and deletions; however, both approaches struggled to adapt to rapid traffic fluctuations. In the case of bursty traffic, the prediction errors caused by the sudden traffic surge led to delayed cell adjustments, resulting in increased average latency and inefficiencies in managing network resources. The proposed method responded quickly to traffic fluctuations through 6P transaction forwarding to higher nodes and traffic-based dynamic cell check period adjustment. This minimized the latency of adding and deleting cells and reduced the average latency compared to the other techniques.

**Figure 19:**
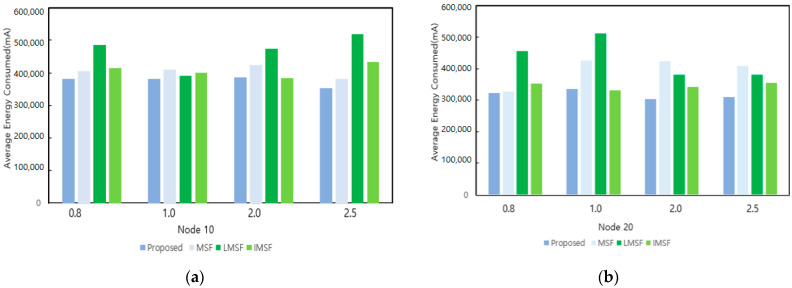
Energy consumption by node changes under bursty traffic. (**a**) Energy consumption as a function of bursty traffic cycles with 10 nodes; and (**b**) energy consumption as a function of bursty traffic cycles at 20 nodes.

Figure 19 shows the comparison of the energy consumption of the proposed technique, conventional MSF, LMSF, and IMSF for different bursty traffic cycles (0.8, 1.0, 2.0, and 2.5) at 10 and 20 nodes, respectively. For a node count of 10, the proposed method achieved the lowest energy consumption during cycles that include bursty traffic, especially when the bursty traffic period was 2.5 days, about 20% lower than the LMSF. Even when the number of nodes was increased to 20, the proposed method maintained the lowest energy consumption. When the bursty traffic period was 1.0, the energy consumption was about 10% lower than the existing method, and the energy efficiency was stable even under increasing traffic. This is because the proposed method efficiently performed cell additions and deletions through the dynamic cell check cycle and 6P transaction forwarding to adapt to traffic changes and optimize network performance quickly. On the other hand, the LMSF and IMSF performed cell addition and deletion predictively. However, due to the prediction error and over-provisioning problem caused by the traffic changes, unnecessary cell negotiation frequently occurred and high energy consumption was recorded.

## 6. Discussion and Limitations

The proposed approach introduces a novel adaptive scheduling mechanism for 6TiSCH networks, designed to enhance the cell allocation efficiency and mitigate packet queue overflow under bursty traffic conditions. By employing a dynamic cell cycle adjustment technique, the method ensures that the cell addition and deletion cycles are adjusted in real time based on the link quality (ETX) and queue utilization (QUF). Furthermore, the 6P transaction forwarding technique allows for proactive negotiation along the routing path, reducing the delays associated with conventional MSF scheduling. These mechanisms collectively enable the network to dynamically respond to traffic fluctuations, minimizing latency and improving packet delivery performance.

Despite these advantages, the proposed approach comes with certain trade-offs and limitations, which must be further explored for broader deployment in real-world IoT environments. Computational overhead in real-time environments: One potential limitation of the proposed technique is the increased computational complexity introduced by continuous queue monitoring and link quality assessment. Unlike the MSF, which follows a fixed scheduling cycle, the dynamic nature of the proposed method requires real-time processing to adjust scheduling decisions. While the simulation results show that the additional overhead remains manageable for small- to medium-scale deployments, its impact on highly resource-constrained IoT devices must be further examined. Future work will explore lightweight implementations to reduce the computational costs while maintaining adaptability. Adaptability to diverse network conditions: The current implementation is optimized for bursty traffic scenarios, where the traffic loads fluctuate significantly over short periods. However, the method has not been fully tested under environments with low, constant traffic loads or highly mobile network topologies. In scenarios where the network conditions change unpredictably—such as in mobile IoT systems or heterogeneous smart city deployments—the effectiveness of the proposed scheduling mechanisms needs further validation. Future research will focus on expanding the adaptability of the model to diverse network environments. Trade-offs between performance and complexity: While the proposed method reduces packet loss and scheduling latency, it introduces an additional control messaging overhead due to the proactive 6P transaction forwarding. This mechanism ensures that the upper-layer nodes anticipate the cell demand in advance, but it may lead to unnecessary negotiation attempts in cases where the traffic variations stabilize or where additional resources are not required.

Further refinement is needed to develop an optimized forwarding threshold mechanism that balances proactive negotiation with protocol efficiency, minimizing unnecessary 6P overheads while maintaining fast response times concerning traffic surges. Future research will focus on evaluating these trade-offs in large-scale deployments, optimizing the method to balance computational efficiency and real-time responsiveness, and exploring potential enhancements, such as reinforcement-learning-based scheduling for dynamic traffic prediction.

## 7. Conclusions

In this study, we proposed a dynamic cell cycle adaptation technique and a 6P transaction forwarding technique to address the limitations of conventional MSF scheduling in 6TiSCH networks, particularly under bursty traffic conditions. The traditional MSF struggles with packet queue overflow and increased latency due to its fixed MAX_NUMCELLS scheduling cycle, which delays necessary cell allocation adjustments. To overcome these limitations, the proposed method incorporates two key enhancements. First, the dynamic cell cycle adaptation technique adjusts the cell addition and deletion cycles in real time based on the link quality (ETX) and queue utilization (QUF), enabling a more responsive adaptation to traffic variations. Second, the 6P transaction forwarding technique allows proactive 6P negotiation requests to be propagated along the routing path, reducing waiting delays and preventing queue overflow during high-traffic periods. The simulation results demonstrate that the proposed approach significantly improves the network performance, achieving over a 20% increase in the packet delivery ratio (PDR) and reducing the average end-to-end latency by more than 10% compared to the MSF, IMSF, and LMSF. Moreover, the method effectively minimizes queue congestion, leading to more efficient cell allocation and enhanced network stability. Scalability tests further confirm that the proposed technique maintains stable performance even in networks with up to 40 nodes, demonstrating its feasibility for large-scale IoT deployments. However, further evaluations are necessary to assess its effectiveness in ultra-large networks exceeding 100 nodes. Despite these improvements, certain limitations remain. While the approach enhances the scheduling responsiveness, it introduces a slight computational overhead due to the real-time monitoring of queue lengths and link quality. Future research will explore machine learning-based adaptive scheduling methods to refine the cell allocation strategies while minimizing the protocol overhead, ensuring efficient operation in highly dynamic and constrained environments. By addressing these challenges, future work will further optimize the proposed technique for energy-efficient, scalable, and high-performance scheduling in 6TiSCH networks. This makes it a promising solution for Industrial IoT, healthcare, and smart city applications, where reliable and adaptive network scheduling is crucial for maintaining seamless communication and operational efficiency.

## Figures and Tables

**Table 1:** Comparison of MSF, LMSF, IMSF, and the Proposed Method for Dynamic Cell Allocation in 6TiSCH Networks.

Technique	Feature	Advantage	Limitations
MSF	Adds and removes cells based on fixed MAX_NUMCELLS cycles.	Simple and easy to implement.	Cannot quickly adapt to burst traffic; may lead to excessive or insufficient cell allocation.
LMSF	Uses a Poisson distribution to predict traffic and dynamically adjusts the number of cells added or removed.	Reduces the number of 6P negotiations, minimizing the network overhead.	Performance heavily depends on the accuracy of the Poisson distribution, making it ineffective for significant traffic variations.
IMSF	Allocates cells based on traffic demand and link quality (ETX) to adjust cell utilization dynamically.	Faster adaptation to traffic fluctuations by using queue occupancy- and ETX-based adjustments.	Risk of over-provisioning due to adding multiple cells at once, leading to inefficient resource utilization.
Proposed Method	Dynamically adjusts cell allocation based on packet queue occupancy and proactively forwards 6P transactions to higher nodes under bursty traffic conditions.	Prevents queue overflow, improves PDR, and minimizes cell allocation delays.	Requires additional computational overhead due to continuous monitoring of queue utilization and ETX.

**Table 2:** Simulation parameters.

Parameter	Value
Simulation platform	6TiSCH
Number of nodes	10, 20, 30, 40
Number of slot frame per run	1200
RPL	OF0
RPL extensions	Unicast
Number of channels	16
Slot frame length	101
App packet period	10
Number of runs	10
Bursty traffic period	0.8, 1.0, 2.0, 2.5
Timeslot length	10 ms
Scheduling function	MSF
Topology	Random
Bursty traffic slot frame	800 to 1000

**Table 3:** Packet delivery ratio (PDR) and latency comparison across different nodes and traffic periods.

Node	Period	PDR (MSF)	PDR (LMSF)	PDR (IMSF)	PDR (Proposed)	Latency (MSF)	Latency (LMSF)	Latency (IMSF)	Latency (Proposed)
10	0.8	82	82	48	85	5.0	3.8	8.0	1.6
	1.0	72	80	73	86	4.55	6.14	5.27	1.5
	2.0	82	83	79	90	3.74	6.27	5.87	1.5
	2.5	86	88	84	93	3.12	4.28	5.26	1.4
20	0.8	72	77	44	85	4.1	4.5	7.6	2.1
	1.0	75	76	59	87	4.34	4.31	7.46	2.3
	2.0	82	83	69	90	3.74	3.87	5.11	2.0
	2.5	85	85	71	91	3.56	2.77	4.12	2.0
40	0.8	53	62	38	75	6.0	4.9	8.7	3.6
	1.0	69	75	43	81	4.67	4.71	7.97	3.5
	2.0	74	77	48	83	4.37	4.37	6.26	3.5
	2.5	79	82	53	87	4.26	4.26	6.23	3.5

**Table 4:** Packet delivery ratio (PDR) and latency comparison across different traffic periods and nodes.

Period	Node	PDR (MSF)	PDR (LMSF)	PDR (IMSF)	PDR (Proposed)	Latency (MSF)	Latency (LMSF)	Latency (IMSF)	Latency (Proposed)
0.8	10	82	82	48	85	5.0	3.8	8.0	1.6
	20	72	77	44	85	4.1	4.5	7.6	2.1
	30	60	52	43	81	5.9	7.53	7.2	2.6
	40	53	62	38	75	6.0	4.9	8.7	3.6

## Data Availability

Data are contained within the article.
